# Se-Methylselenocysteine Inhibits Apoptosis Induced by Clusterin Knockdown in Neuroblastoma N2a and SH-SY5Y Cell Lines

**DOI:** 10.3390/ijms151121331

**Published:** 2014-11-18

**Authors:** Chao Wang, Zhenyu Zeng, Qiong Liu, Renli Zhang, Jiazuan Ni

**Affiliations:** 1 Changchun Institute of Applied Chemistry, Chinese Academy of Sciences, Changchun 130022, China; E-Mail: raulw2003@163.com; 2 Shenzhen Key Laboratory of Marine Biotechnology and Ecology, College of Life Sciences, Shenzhen University, Shenzhen 518060, China; E-Mail: tim@alevel.com.cn; 3 University of Chinese Academy of Sciences, Chinese Academy of Sciences, Beijing 100049, China; 4 Microorganism Examination Division, Shenzhen Center for Disease Control and Prevention, Shenzhen 518055, China; E-Mail: renlizhangszcdc@aliyun.com

**Keywords:** clusterin (Clu), Se-methylselenocysteine (MSC), cell apoptosis, RNA interference (RNAi), gene knockdown

## Abstract

Apoptosis, as a programmed cell death process, is essential for the maintenance of tissue function in organisms. Alteration of this process is linked to many diseases. Over-expression of clusterin (Clu) can antagonize apoptosis in various cells. Selenium (Se) is an essential trace element for human health. Its biological function is also associated with cell apoptosis. To explore the function of Clu and the impact of Se in the process of apoptosis, several short-hairpin RNAs (shRNA) were designed for the construction of two sets of recombinant plasmids: one set for plasmid-transfection of mouse neuroblastoma N2a cells (N2a cells); and the other set for lentiviral infection of human neuroblastoma SH-SY5Y cells (SH-SY5Y cells). These shRNAs specifically and efficiently interfered with the intracellular expression of Clu at both the mRNA and protein levels. The *Clu*-knockdown cells showed apoptosis-related features, including down-regulation of antioxidative capacity and the Bcl-2/Bax ratio and up-regulation of caspase-8 activity. Se-methylselenocysteine (MSC) at an optimum concentration of 1 μM could reverse the alteration in antioxidative capacity, Bcl2/Bax ratio and caspase-8 activity caused by *Clu*-knockdown, thus inhibiting apoptosis and maintaining cell viability. The results hereby imply the potentiality of Clu and Se in neuroprotection.

## 1. Introduction

Apoptosis is a controlled cell death process, which plays important roles in many physiological processes, such as embryogenesis, metamorphosis, cellular homeostasis, and as a defensive mechanism to eliminate infected, damaged or mutated cells [1]. Apoptosis of tumor cells is beneficial because it can terminate the cells growth and decrease the metastasis of tumors. However, excessive and inappropriate cell death can cause neurological diseases, such as Alzheimer’s disease (AD) and Parkinson’s disease (PD). Apoptosis is mainly mediated by two protein families, the caspases family and the Bcl-2 family.

Clusterin (Clu), also named TRPM-2, GP-80, pADHC-9, SP40/40, CLI and ApoJ, is first isolated from ram rete testes fluid [2]. It is constitutively secreted by several types of cells and found in all human body fluids [3]. Clu plays important roles in various pathophysiological processes, such as tissue remodeling, sperm maturation, lipid transport, complement regulation and cell apoptosis [3].

The* Clu* gene has three different transcripts, which are translated into several distinct protein isoforms with different cellular localization [4]. Among these, the secreted Clu (s-Clu) is a 75–80-kDa glycoprotein, composed by a 40-kDa α-chain and a 40-kDa β-chain [5]. The nuclear Clu (n-Clu) is the protein translocated from the cytoplasm to the nucleus. n-Clu has been recently found as a 55-kDa protein in MCF-7 cells [6], which is originated from a 49-kDa cytoplasm Clu (c-Clu) after apoptosis induction. c-Clu is translated from an alternatively spliced Clu transcript [7].

Clu was initially found to be associated with cell death in the regressing rat ventral prostate [8]. However, recent studies suggest that Clu plays a dual role in apoptosis. s-Clu is considered as cytoprotective and may be involved in the clearance of cellular debris and the promotion of phagocytosis [2]. n-Clu, on the other hand, is a pro-death protein that induces apoptosis [9]. Several cytokines, including transforming growth factor β (TGF-β) and tumor necrosis factor α (TNF-α), can induce over-expression of Clu in various cell types to overcome apoptosis [10]. Clu protects H9c2 cardiomyocytes from oxidative stress-induced apoptosis via the Akt/GSK-3β signaling pathway [11]. Clu inhibits mitochondrial apoptosis by suppressing p53-activating stress signals and stabilizing the cytosolic Ku70–Bax protein complex [12]. It may also promote oncogenic transformation and tumor progression by interacting with activated Bax [13].

Selenium (Se) is an essential trace element for human health. Se depletion may lead to various diseases, such as cancer, immune dysfunction, reproductive disorders and neurodegenerative diseases [14]. Monomethylated forms of Se, especially Se-methylselenocysteine (MSC), are precursors of methylselenol, which have potent cancer chemopreventive activity [15,16]. It has been reported that MSC protects human hepatoma cells against oxidative stress [17]. MSC prevents oxidative damage induced by irradiation in spleen [18] and in rat lungs [19] by reinforcing antioxidant capacity. MSC and vitamin E show a synergistic effect in ameliorating acute ethanol-induced oxidative damage in rat [20]. However, the relationship between MSC and cell apoptosis is still not very clear.

In this study, several recombinant plasmids were constructed to specifically and efficiently decrease the intracellular expression of Clu in two types of neuroblastoma cells, N2a and SH-SY5Y cells, which are generally used as the cell lines for neuronal study. Apoptosis was found in the *Clu*-knockdown cells, and MSC was found to inhibit apoptosis.

## 2. Results

### 2.1. RNAi Silencing the Clu Gene Expression in the N2a and SH-SY5Y Cells

A number of recombinant plasmids for interfering with Clu expression were successfully constructed and confirmed by sequencing. Plasmids pGPU–*Clu*–mus-488 (sh488), pGPU–*Clu*–mus-577 (sh577), pGPU–*Clu*–mus-644 (sh644) and pGPU–*Clu*–mus-1535 (sh1535) were individually used for interfering with Clu expression in N2a cells. Plasmids pLKO–shRNAClu1 (Clu-1) and pLKO–shRNAClu2 (Clu-2) were constructed for interfering with Clu expression in SH-SY5Y cells. Plasmids pGPU–Clu–mus–shSc (Sc) and pLKO–shRNANc (Nc) were constructed for the N2a cells and the SH-SY5Y cells, respectively, as the corresponding controls of the RNAi assay.

In order to get efficient transfection, different ratios of Sc plasmid and lipofectamine 2000, including 1:0.5, 1:1, 1:2, 1:3, 1:4, 1:5 (μg/μL), were made for plasmid transfection to the N2a cells. According to the green fluorescence intensity shown in the transfected cells, the best ratio of plasmid and lipofectamine 2000 was 1:1 (data not shown), and this ratio was selected for the following plasmid transfection experiments.

**Figure 1:**
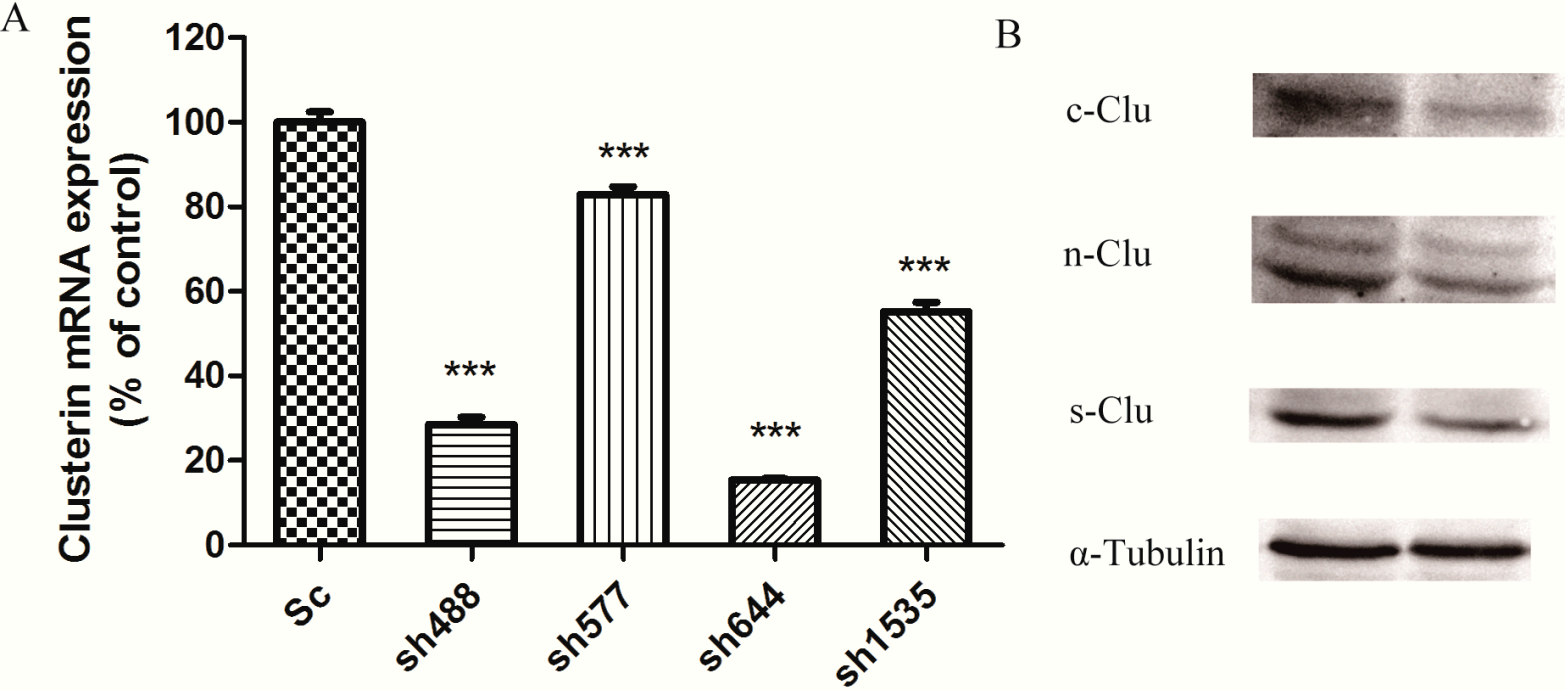
Knockdown of *Clu* expression in N2a cells by RNA interference. (**A**) Down-regulation of *Clu* mRNA expression by the recombinant plasmids, including pGPU–*Clu*–mus-488 (sh488), pGPU–*Clu*–mus-577 (sh577), pGPU–*Clu*–mus-644 (sh644), pGPU–*Clu*–mus-1535 (sh1535) and pGPU–*Clu*–mus–shSc (Sc), respectively. The mRNA expression level was detected by the real-time PCR assay; (**B**) Decreased protein expression level of Clu in N2a cells by RNAi with the pGPU–*Clu*–mus-644 (sh644) construct. Protein expression was detected by western blot analysis. The construct pGPU–*Clu*–mus–shSc (Sc) was used for the control cells. *** *p* < 0.001* vs.* the control. c-Clu, cytoplasmic Clu; n-Clu, nucleic Clu; s-Clu, secreted Clu.

To interfere with the expression of Clu in N2a cells, the recombinant plasmids sh488, sh577, sh644, and sh1535, along with the control plasmid Sc, were used for cell transfection. After 72 h of incubation with the transfection reagent, cells were collected to extract RNA for real-time PCR. β-Actin was used as a standard to normalize the expression level of Clu in different samples. The relative quantity of the target gene was estimated by the formula 2^−ΔΔ*C*t^, where *C*_t_ is the number of cycles required for the fluorescent signal to cross the threshold, Δ*C*_t_ = *C*_t_(Clu) − *C*_t_(β-actin) and ΔΔ*C*_t_ =Δ*C*_t_(RNAi group) − Δ*C*_t_(Sc group). Real-time PCR results showed that all four RNAi recombinant plasmids significantly down-regulated the expression of Clu in the N2a cells (Figure 1A). The sh644 plasmid had the highest inhibition efficiency, which reduced Clu expression by 84.58% (*p* < 0.001). The inhibition efficiency of plasmids sh488, sh577 and sh1535 were 71.44%, 17.08% and 44.76%, respectively. Thus, sh644 was selected as the interfering plasmid for the following research.

After transfecting plasmid sh644 to N2a cells, the cell extract was separated to get total proteins for the western blot analysis, using α-tubulin as an internal standard. Western blot results showed that plasmid sh644 also significantly decreased the expression of Clu at the protein level (Figure 1B). All different isoforms of Clu,* i.e.*, s-Clu, n-Clu and c-Clu, are down-regulated by sh644 plasmid transfection.

**Figure 2:**
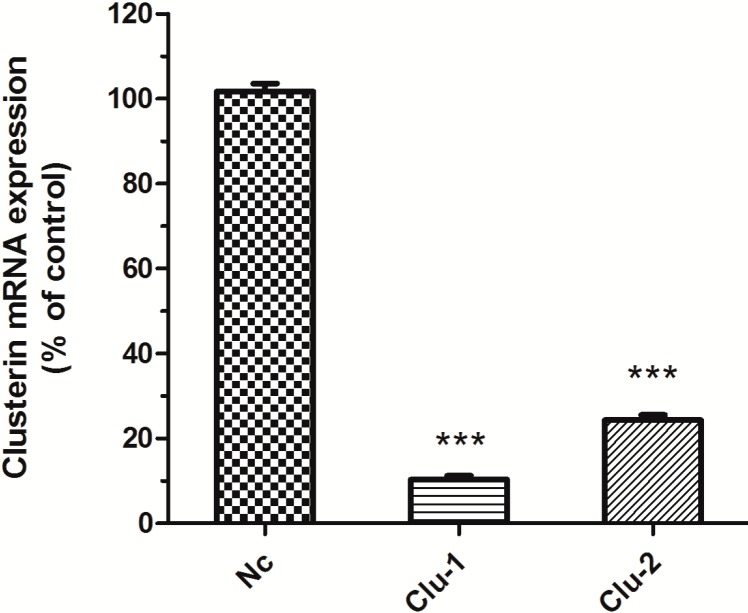
Knockdown of *Clu* gene expression in SH-SY5Y cells. The cells were infected with lentivirus packaged from the plasmids pLKO–shRNAClu1 (Clu-1), pLKO–shRNAClu2 (Clu-2) and pLKO-shRNANc (Nc), respectively. The mRNA expression level was detected by the real-time PCR assay. Cells infected with the lentivirus containing the pLKO–shRNANc plasmid (Nc) were used as the control. *** *p* < 0.001* vs.* the control.

The packaged lentivirus was used to interfere with Clu expression in SH-SY5Y cells. Three recombinant plasmids, Clu-1, Clu-2 and Nc, were successfully constructed, which are respectively co-transfected HEK293T cells with the packaged plasmids pCMV–VSV-G and pCMV-delta8.9. The packaged lentivirus were used to infect SH-SY5Y cells to knockdown Clu expression. Puromycin (0.5 μg/mL) was used to screen the infected cells for 10 days. The medium with puromycin was changed every 2–3 days. Total RNA of the infected cells was extracted for real-time PCR analysis to detect *Clu* expression. Results showed that lentivirus packaged from both Clu-1 and Clu-2 plasmids significantly decreased the expression of *Clu* (Figure 2). The expression level of *Clu* was significantly reduced by 88.99% (*p* < 0.001) by the lentivirus packaged from the Clu-1 plasmid. The inhibition efficiency of plasmids Clu-2 is 66.25% (*p* < 0.001). Thus, the Clu-1 plasmid was selected as the interfering plasmid for the following research.

### 2.2. Altered Expression Levels of Apoptosis-Related Proteins in the Clu-Knockdown Cells

**Figure 3:**
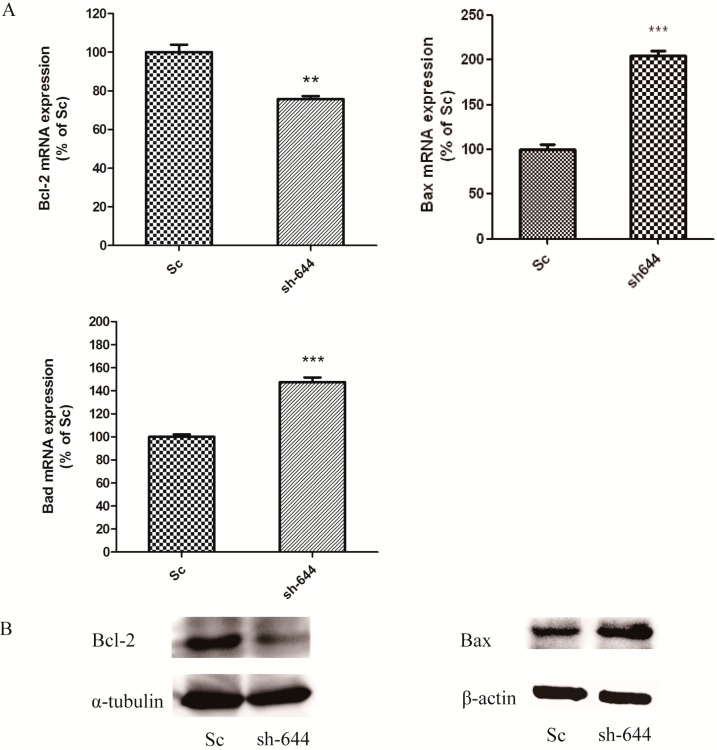
Altered expression levels of apoptosis-related genes *Bax*, *Bcl-2* and *Bad* in the *Clu*-knockdown N2a cells. (**A**) Real-time PCR detection of *Bax*, *Bcl-2* and *Bad* mRNA expression; (**B**) Western blot analyses of Bax and Bcl-2 protein expression. Cells transfected with pGPU–*Clu*–mus–shSc (Sc) were used as the control. ** *p* < 0.01 and *** *p* < 0.001* vs.* the control.

Bax, Bcl-2 and Bad belong to the family of Bcl-2 proteases, which mediate cell apoptosis. In the *Clu*-knockdown N2a cells, altered levels of Bax, Bcl-2 and Bad were detected at both mRNA and protein levels by real-time PCR and western blot analyses, respectively. β-Actin served as an internal standard. At the mRNA level, the expression of *Bcl-2* decreased to 75.69% (*p* < 0.01), while the expression of *Bax* and *Bad* increased to 202.67% (*p* < 0.001) and 147.46% (*p* < 0.001) respectively, compared to the Sc control (Figure 3A). At the protein level, the expression of Bcl-2 was also significantly decreased (Figure 3B), and the expression of Bax was significantly increased (Figure 3B). The results from both mRNA and protein levels showed the same trends in the alteration of apoptosis-related factors.

**Figure 4:**
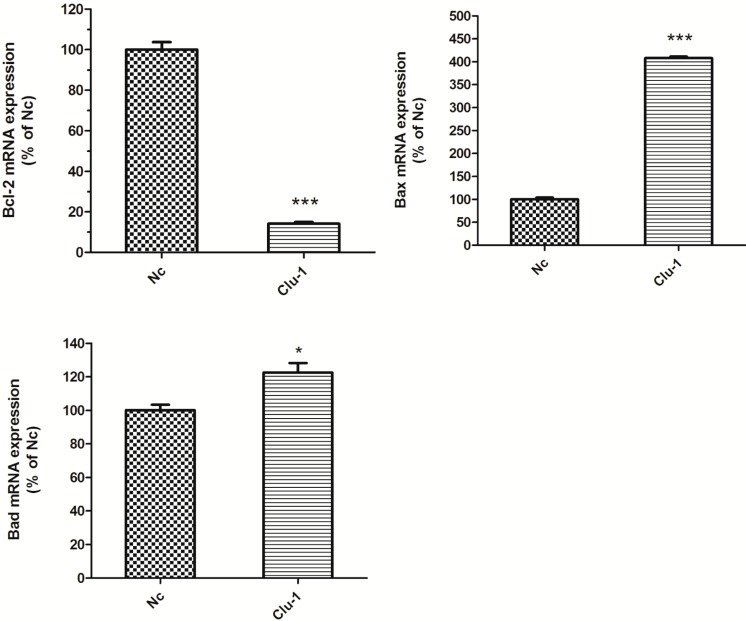
Altered expression levels of apoptosis-related genes *Bax*, *Bcl-2* and *Bad* in the *Clu*-knockdown SH-SY5Y cells. Real-time PCR was used to detect the mRNA expression level. Cells infected with the lentivirus containing pLKO–shRNANc (Nc) served as the control. * *p* < 0.05 and *** *p* < 0.001* vs.* the control.

In the *Clu*-knockdown SH-SY5Y cells, the mRNA expression levels of *Bax*, *Bcl-2* and *Bad* were detected by real-time PCR. The level of *Bcl-2* was decreased to 14.12% (*p* < 0.001), while the levels of *Bax* and *Bad* were increased to 407.73% (*p* < 0.001) and 122.53% (*p* < 0.05) respectively, compared to the Nc control (Figure 4). The expression levels of *Bax*, *Bcl-2* and *Bad* changed in the same way in the *Clu*-knockdown N2a and SH-SY5Y cells.

### 2.3. Effects of MSC on Cell Viability and Apoptosis-Related Proteins in the Clu-Knockdown Cells

Cell viability was altered in the *Clu*-knockdown cells treated with different concentrations of MSC. The optimum concentration of MSC was selected according to its effect on cell viability. After being transfected with the plasmid sh644 for 48 h, cells were dissociated and counted to seed into 96-well plates with 5 × 10^3^ cells in 100 μL of medium per well. MSC at a series of concentrations (0.1, 0.5, 1.0, 2.0 and 3.0 μM) was added into plate wells, respectively, to treat the transfected cells for another 24 h. Cell viability was then detected by the CCK-8 assay.

**Figure 5:**
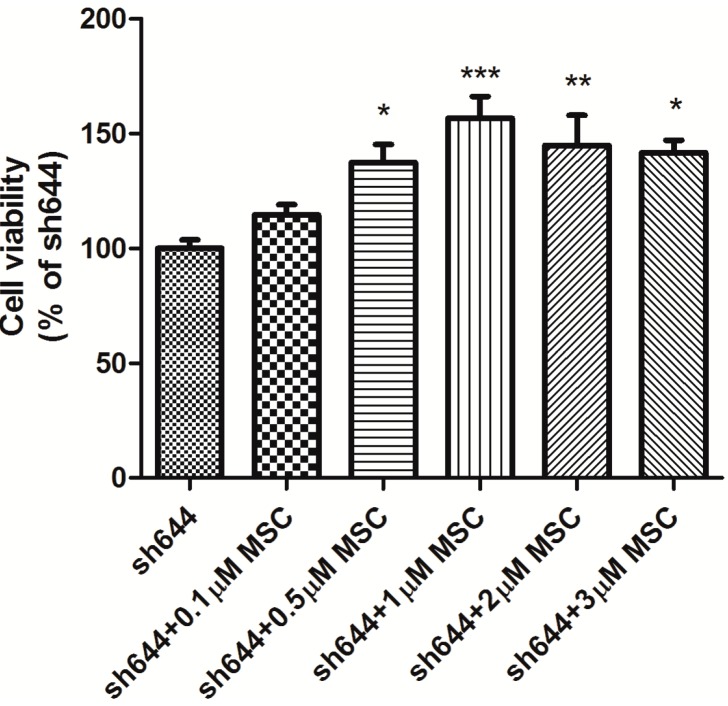
Dose-dependent effect of Se-methylselenocysteine (MSC) on the viability of *Clu*-knockdown N2a cells. Cells transfected with the plasmid pGPU–*Clu*–mus–sh644 (sh644) were used as the control. * *p* < 0.05, ** *p* < 0.01 and *** *p* < 0.001* vs.* the control.

The results showed that all concentrations of MSC could increase the viability of *Clu*-knockdown N2a cells; among them, 1 μM of MSC is the optimum in up-regulating cell viability. With the treatment of 1 μM MSC, the cell viability improved by 1.57 times (*p* < 0.001) (Figure 5).

**Figure 6:**
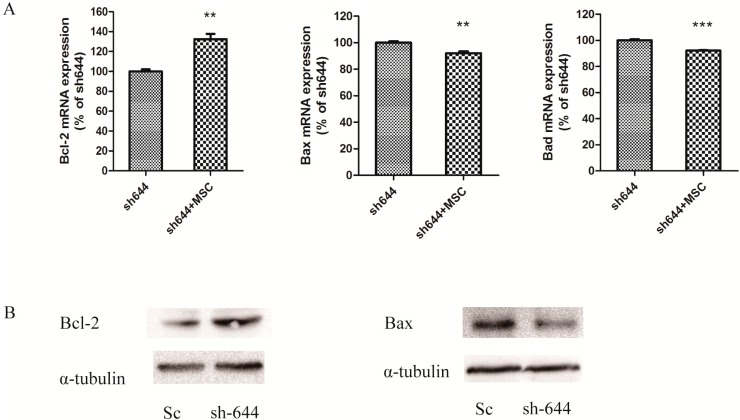
Altered expression levels of apoptosis-related genes *Bax*, *Bcl-2* and *Bad* in the *Clu*-knockdown N2a cells treated with 1 μM MSC. Cells transfected with the plasmid pGPU-*Clu*-mus-sh644 (sh644) were used as the control. (**A**) Real-time PCR detection of *Bax*, *Bcl-2* and *Bad* mRNA expression; (**B**) Western blot analyses of Bax and Bcl-2 protein expression. ** *p* < 0.01 and *** *p* < 0.001* vs.* the control.

The effects of MSC on apoptosis-related proteins in the *Clu*-knockdown cells were also investigated. After transfecting plasmid sh644 for 48 h in N2a cells, MSC (1 μM) was added into the culture medium to treat cells for another 24 h. Then, the cells were collected and extracted for RNAs and proteins for real-time PCR and western blot analyses. At the mRNA level, the expression level of *Bcl-2* was increased to 132% (*p* < 0.01), while the levels of *Bax* and *Bad* were decreased to 91% (*p* < 0.01) and 90% (*p* < 0.001), respectively, compared to the sh644 plasmid-transfected cells (Figure 6A). Obviously, MSC also increased Bcl-2 expression and decreased Bax expression at protein levels (Figure 6B).

### 2.4. Effects of MSC on Total Antioxidant Capacity and Caspase-8 Activity in the Clu-Knockdown Cells

**Figure 7:**
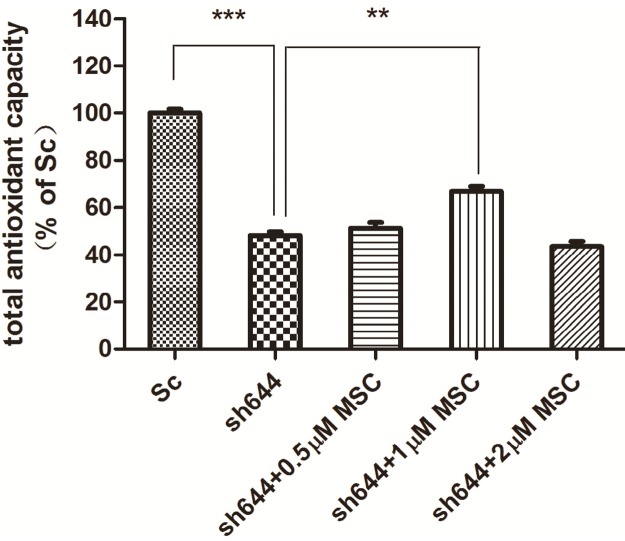
Total antioxidant capacity of the *Clu*-knockdown N2a cells treated with MSC. *** *p* < 0.001* vs.* the cells transfected with the plasmid pGPU–*Clu*–mus–Sc (Sc). ** *p* < 0.01* vs.* the cells transfected with the plasmid pGPU–*Clu*–mus–sh644 (sh644).

MSC at different concentrations (0.5, 1.0 and 2.0 μM) was added into wells to treat the transfected N2a cells for another 24 h, after transfection of plasmids sh644 and Sc, respectively, to the N2a cells for 48 h. Total proteins were extracted from the cells, and the total antioxidant capacity was detected. Compared to the Sc control, the total antioxidant capacity of the *Clu*-knockdown cells (sh644) was reduced to 48.12% (*p* < 0.001) (Figure 7). Treatment with MSC could inhibit, to some extent, the decrease of total antioxidant capacity caused by *Clu*-knockdown. The optimum concentration of MSC is 1 μM, which increased the total antioxidant capacity by 1.4-times (*p* < 0.01) (Figure 7), compared to the sh644 group. Therefore, *Clu*-knockdown resulted in the decrease of total antioxidant capacity in N2a cells, while this decrease could by reversed by the treatment of 1 μM MSC.

**Figure 8:**
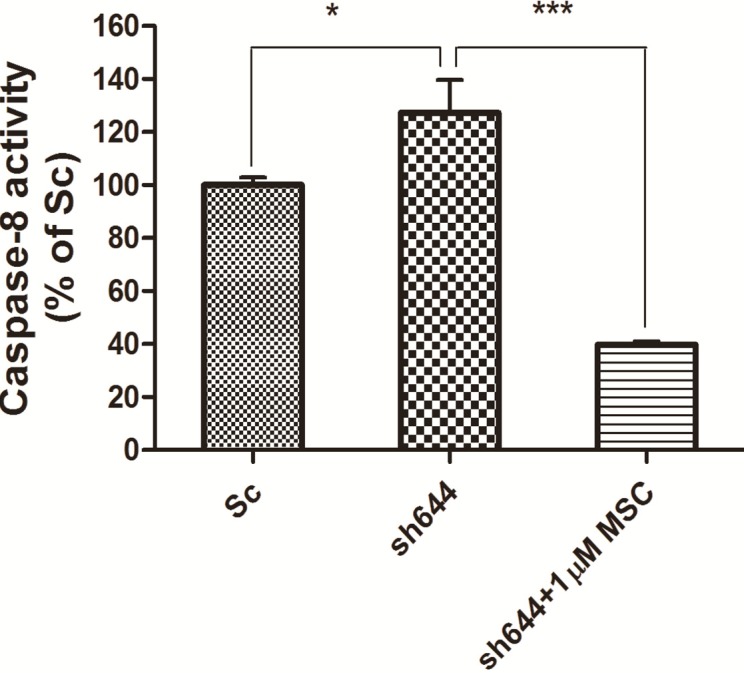
Altered caspase-8 activity in *Clu*-knockdown N2a cells treated with 1 μM MSC. * *p* < 0.05* vs.* the cells transfected with the plasmid pGPU–*Clu*–mus–Sc (Sc). *** *p* < 0.001* vs.* the cells transfected with the plasmid pGPU–*Clu*–mus–sh644 (sh644).

Synchronously, caspase-8 activity was measured for those cells transfected with the plasmid sh644 and treated with 1 μM MSC, along with the Sc plasmid-transfected cells. Compared to the Sc control, the caspase-8 activity of the *Clu*-knockdown N2a cells (sh644 group) was increased to 127.24% (*p* < 0.05) (Figure 8). However, caspase-8 activity of the *Clu*-knockdown cells was decreased to 31% (*p* < 0.001) (Figure 8) after the treatment of 1 μM MSC, compared to the sh644 group. Those results suggested that 1 μM MSC could inhibit the up-regulated caspase-8 activity caused by *Clu-*knockdown in N2a cells.

## 3. Discussion

Apoptosis is a process of the spontaneous death of normal cells* in vivo* induced by physiological and pathological stimuli. It is an active, highly ordered, genetic control process, with a series of enzymes involved [21]. Apoptosis plays a critical role in both the physiological process and the pathological process of diseases, including cancer and neurodegenerative diseases. Clu is closely associated with apoptosis. Clu can reduce apoptosis by binding to the activated Bax and sequestering it in the cytoplasm, thus preventing Bax from entering mitochondria and causing cytochrome c release, eventually triggering apoptosis [13]. The anti-apoptotic mechanism of Clu has been reported to down-regulate Ku70 acetylation by directly blocking Bax activation in neuroblastoma [22]. Clu can inhibit NF-κB signaling through stabilization of IκBs (inhibitor of κB) [23]. Over-expression of human Clu has been reported to protect human epidermal keratinocyte (HaCaT) cells from ropivacaine-induced apoptosis [24]. MSC is a new type of organic selenium compound, which is prevalent in broccoli [25] and garlic [26]. MSC has lots of advantages among selenium compounds to become an additive in nutrition and medicine, such as low toxicity, clear and stable molecular structure and well-studied metabolic mechanism in the human body [18]. As an organic form of the selenium compound, MSC could recover the activities of antioxidant enzymes (such as GSH-Px and SOD) and reduce the levels of malondialdehyde (MDA) and carbonyl protein (CP) [20]. In this study, low concentrations of MSC were used to study the relationship between MSC and Clu in terms of cell apoptosis altered by *Clu*-knockdown.

The expression levels of Clu in N2a and SH-SY5Y cells were significantly down-regulated by RNAi assays in this paper. *Clu*-knockdown in those cells significantly up-regulated the expression of pro-apoptosis genes *Bax* and *Bad* and the enzyme activity of caspase-8 and down-regulated the expression of anti-apoptosis gene *Bcl-2*, the total antioxidant capacity and cell viability. Further studies showed that 1 μM MSC could reverse those changes to some extent, thus protecting cells from apoptosis induced by *Clu*-knockdown.

In the present study, *Clu* knockdown was found to cause the apoptosis of AD cells, which corresponds to previous reports that Clu plays a role in antagonizing oxidative stress and oxidant injury [11,27]. Besides, Clu has also been reported to protect H9c2 cardiomyocytes against H_2_O_2_ stimulus [11], inhibit ROS generation in human retinal pigment epithelial cells [28] and protect human corneal endothelial cells from oxidative injury-mediated cell death via inhibition of ROS production [29]. Thus, *Clu*-knockdown can decrease the total antioxidant capacity due to the role of Clu in maintaining redox equilibrium* in vivo*. The addition of MSC increased the antioxidative capacity of AD cells, thus preventing the apoptosis caused by *Clu* knockdown.

Apoptosis is mainly mediated by two proteases families,* i.e.*, the Bcl-2 family [30] and the caspases family [31]. The Bcl-2 family includes both pro-apoptosis genes, such as Bax and Bad, and anti-apoptosis genes, such as Bcl-2 and Bcl-xL. Bcl-2 and Bax can be formed either as homodimers or heterodimers. Bax homodimers induce apoptosis. If the Bcl-2 expression increases, Bcl-2 and Bax can form the Bax–Bcl-2 heterodimer, which is more stable than the Bax–Bax homodimer, to inhibit cell apoptosis induced by Bax–Bax homodimer. Thus, the intracellular ratio of Bcl-2/Bax can regulate the process of cell apoptosis [30]. Bad can replace Bax from the Bcl–xL–Bax dimmer to increase the formation of the Bax–Bax homodimer, leading to the induction of apoptosis [32]. In this study, *Clu*-knockdown resulted in the reduction of the Bcl-2/Bax ratio and promotion of the Bad level; thus, more Bax–Bax homodimers were formed and caused cell apoptosis. MSC at low concentrations could reverse the changes of *Bcl-2*, *Bax* and *Bad* expression levels, therefore inhibiting apoptosis induced by *Clu*-knockdown.

The caspase family also plays very important roles in apoptosis. The caspases-mediated apoptosis pathways are usually activated by a death receptor signal, causing the upstream pro-caspase-8 activation, which results in the activation of caspase-9 and caspase-3 [33]. Caspase-8 is one of the most important proteins in the caspase family, which activates almost all downstream caspases, including caspase-3, caspase-7, caspase-4, caspase-9 and caspase-10, to induce apoptosis. The activity of caspase-8 can be inhibited by cytokine response modifier A (CrmA) as a negative regulation [34]. In the present study, *Clu*-knockout caused the activation of caspase-8, which induced a series of cascade reactions leading to apoptosis. MSC at low concentrations reduced the activation of caspase-8 and, thus, inhibited cell apoptosis.

Summarily, the endogenous Clu and supplemented MSC play important roles in preventing N2a and SH-SY5Y cells from entering apoptosis by maintaining antioxidant capacity, balancing Bcl-2 and Bax proportion and regulating caspase-relevant signal pathways. Clu is an enigmatic molecule associated with various physiological processes and diseases. Recently, large-scale population surveys have shown that Clu is linked to AD [35,36]. Clu regulates the generation and clearance of β-amyloid protein (Aβ), which is one dominant feature and cause of AD [37]. Compared to WT mice, the levels of Clu in serum from triple transgenic (*PS1_M146V_*/*APP_Swe_*/*Tau_P301L_*) AD mice are decreased [38]. In this paper, we showed that a decrease of Clu leads to the apoptosis of neurocytes, which could be restored by SMC. Neurocyte apoptosis also occurs in AD and is an important cause of this disease. Results in this paper thus imply that the optimum expression level of Clu is essential for the growth of neurocytes, and selenium supplementation at proper doses helps to prevent neurocytes from the apoptosis induced by Clu down-regulation. This paper also provides an effective RNA interfering method for *Clu* knockdown and for the study of SMC in preventing Clu-relevant cell apoptosis, which implies the potentiality of SMC in maintaining the expression level of Clu in the brain and the treatment of AD.

## 4. Experimental Section

### 4.1. Materials

N2a and SH-SY5Y cells were purchased from the Cell Bank of Type Culture Collection of the Chinese Academy of Sciences (Shanghai, China). Human embryonic kidney 293T (HEK293T) cells and *E. coli* strain Top10 were kept in our lab. Clu polyclonal and α-tubulin monoclonal antibodies were purchased from Santa Cruz (Dallas, TX, USA). β-Actin monoclonal antibody was purchased from Abcam (Cambridge, MA, USA). Bax, Bcl-2 monoclonal antibodies, the caspase-8 activity assay kit, total antioxidant capacity assay kit and cell counting kit (CCK-8) were purchased from Beyotime (Guangzhou, China). Lipofectamine 2000 for transfection was from Invitrogen (Grand Island, NY, USA). Se-methylselenocysteine was purchased from Sigma (St. Louis, MO, USA).

### 4.2. Mammalian Cell Culture and Viability Assay

HEK293T cells were cultured in Dulbecco’s Modified Eagle’s Medium (DMEM) supplemented with 10% fetal bovine serum (FBS). N2a cells were maintained in a 1:1 mixture of DMEM (containing 10% FBS) and Opti-MEM I (Gibco, Grand Island, NY, USA). SH-SY5Y cells were grown and treated in DMEM/F12 (1:1) media containing 10% FBS and 2 mM l-glutamine. All media were supplemented with 100 U/mL penicillin and 100 µg/mL streptomycin, and cells were cultured at 37 °C in 5% CO_2_.

Cell viability was measured using the CCK-8 assay. After plasmid transfection for 48 h, cells were dissociated by trypsin and counted through the trypan blue assay. Those cells were then seeded into 96-well plates at about 5 × 10^3^ cells per well in 100 μL of medium. MSC were added into wells, and cells were cultured for another 24 h. Ten microliters of CCK-8 reagent were added to each well, and the cells were incubated at 37 °C for another 2 h. The absorbance at 450 nm was measured with a reference wavelength at 650 nm using a SpecTRA MAX 190 microplate reader [39].

### 4.3. Plasmid Construction

According to the mRNA sequence of mouse *Clu* (Accession No. NM_013492.2), small interfering RNA (siRNA) for down-regulating *Clu* gene expression was designed using the program RNAi Designer (Invitrogen, Grand Island, NY, USA). The nucleotide sequences and targeted regions of four siRNAs are listed in Table 1. Four short hairpin RNAs (shRNA) were designed according to the corresponding siRNAs. Each shRNA consisted of an AgeI cutting site, the sequence of siRNA, a linker (TTCAAGAGA), the reversed complementary sequence of siRNA, a RNA polymerase III transcription terminator (TTTTTT) and a EcoRI cutting site. The sense and antisense strands of each shRNA were synthesized separately (the Shanghai Genepharma Company, Shanghai, China), annealed, cut by two restriction endonucleases and inserted into the plasmid pGPU–GFP–Neo. These recombinant plasmids, including pGPU–*Clu*–mus-488 (sh488), pGPU–*Clu*–mus-577 (sh577), pGPU–*Clu*–mus-644 (sh644) and pGPU–*Clu*–mus-1535 (sh1535), were constructed to interfere with mouse *Clu* expression in N2a cells. The plasmid pGPU–*Clu*–mus–shSc (Sc) was constructed for the control cells.

Another two shRNAs (Table 2) were similarly designed and synthesized to target human *Clu* gene (Accession No. NM_001831.3), which were consistent with a previous report [3]. After annealing, the shRNA strands were inserted into plasmid PLKO.1-puro. Those recombinant plasmids, including pLKO–shRNAClu1 and pLKO–shRNAClu2, were constructed for packaging lentivirus, respectively, and used to interfere with Clu expression in SH-SY5Y cells. The plasmid pLKO–shRNANc (Nc) was constructed to package lentivirus for the control cells.

**Table 1:** Specific siRNA targeted region for mouse *Clu*.

No.	siRNA Targeted Sequence (5'–3')	Targeted Region
1	GGACACTAGGGATTCTGAAAT	488–508
2	GCCTGAAGCATACCTGCATGA	577–597
3	GCAGCTAGAGGAGTTTCTAAA	644–664
4	GGAGAAGGCGCTACAGGAATA	1535–1555

**Table 2:** Specific siRNA targeted region for human *Clu*.

No.	siRNA Targeted Sequence (5'–3')	Targeted Region
1	CCAGAGCTCGCCCTTCTAC	434–453
2	GTCCCGCATCGTCCGCAGC	665–684

### 4.4. Plasmid Transfection and Drug Treatment

Lipofectamine 2000 was used to transfect plasmids into cells. Before transfection, the full culture medium was changed to serum-free medium. Plasmids and the transfection reagent, lipofectamine 2000, were both diluted with Opti-MEM I and then mixed thoroughly. The mixture prepared was left at room temperature for 30 min, added into the cell culture with serum-free medium and incubated for 4 h at 37 °C in 5% CO_2_. Then, the serum-free medium was changed to full medium, and the cells were cultured for another 48 h. Different concentrations of MSC (0.1, 0.5, 1.0, 2.0 and 3.0 µM) were prepared to treat the transfected cells for another 24 h before cell harvest.

### 4.5. Lentivirus Packaging and Cell Infection

The package system for the lentivirus contains three plasmids: the lentiviral interference vector PLKO.1-puro, the package plasmids pCMV–VSV-G and pCMV-delta8.9. HEK293T cells were used to package the lentivirus. HEK293T cells in the logarithmic growth phase were seeded into a 60-mm plate. Once cell fusion reached 80%, the recombinant lentiviral frame plasmid pCMV-delta8.9 and the packaging helper plasmid pCMV–VSV-G were co-transfected into HEK293T cells with the aid of lipofectamine 2000. The supernatant of HEK293T culture was collected and centrifuged (3000 rpm) at 4 °C for 5 min to remove cell debris after 48 h of transfection. The packaged lentiviruses were in the supernatant.

SH-SY5Ycells were seeded in 6-well plates (10^6^ cells per well) and grown to approximately 50% cell fusion after 12 h of cultivation. The virus solution was diluted 10-fold with complete cell culture medium, and polybrene was added to a final concentration of 5 μg/mL. The medium in 6-well plate was replaced with the fresh prepared virus diluted medium. Puromycin (0.5 μg/mL) was used to screen the infected cells for 10 days. The medium with puromycin was changed every 2–3 days.

### 4.6. Real-Time PCR Analysis

Total RNA was isolated using the RNAiso Plus Kit (Takara, Dalian, China) according to the supplier’s instructions. RNA was quantified by optical density measurements at 260 and 280 nm. RNA integrity was confirmed by running samples on 1% agarose gel. On microliter of total RNA was added to a 10-μL reaction mixture of the PrimeScript™ RT reagent Kit (Takara, Dalian, China), according to the supplier’s instructions. The reverse transcription products cDNA were stored at −70 °C until assay.

Gene-specific primers (Table 3) and the SYBR Premix Ex Taq™ (Takara, Dalian, China) were used for real-time PCR quantification. β-Actin served as the standardized control. All cDNA samples were diluted 5-fold with RNase-free water before being used as templates in quantity analysis. The 20-μL reaction mixture contained 2 μL cDNA, 0.4 μL each primer (10 μM), 10 μL of SYBR^®^ Premix Ex Taq™, 0.4 μL ROX Reference Dye II and 6.8 μL PCR-grade water. The RT-PCR protocol was as follows: 1 min initial denaturation at 95 °C, 40 cycles of 95 °C for 5 s and 60 °C for 30 s. To verify the specificity of each primer, a melting-curve analysis was included. The relative quantity of the target gene was estimated by the 2^−ΔΔ*C*t^ method after normalizing to the expression level of β-actin.

**Table 3:** Primer sequences used for real-time RT-PCR detection.

Gene	Primer	Primer Sequences (5'–3')
*Clu*	F_Clu_	GCCAGGAGCTGAACGACT
	R_Clu_	GGGATGAGGTGTTGAGCACT
*β-actin*	F_β-actin_	CGGGAAATCGTGCGTGAC
	R_β-actin_	CAGGAAGGAAGGCTGGAAGAG
*Bax*	F_Bax_	GCCAGCAAACTGGTGCTCAA
	R_Bax_	ATGTCCAGCCCATGATGGTTC
*Bad*	F_Bad_	GAGTGACGAGTTTGTGGACTCCT
	R_Bad_	CAAGTTCCGATCCCACCAG
*Bcl-2*	F_Bcl-2_	CATGTGTGTGGAGAGCGTCAA
	R_Bcl-2_	GCCGGTTCAGGTACTCAGTCA

### 4.7. Western Blot Analysis

Cells transfected with plasmids sh644 or Sc were lysed by RIPA lysis buffer in the presence of proteinase inhibitors. Fifty micrograms of protein were submitted to SDS-PAGE using a 10% gel and transferred onto polyvinylidene difluoride (PVDF) membranes. Immunoblotting was performed with a goat antibody, against the rat Clu (sc-6419, Santa Cruz, Dallas, TX, USA) and an appropriate secondary antibody. Blots were developed using the ECL system (Pierce, Rockford, IL, USA) and exposed to autoradiography. β-Actin or α-tubulin was used as an internal standard to control equal loading of total proteins.

### 4.8. Caspase-8 Activity Assay

Caspase-8 activity was measured using the caspase activity kit, according to the manufacturer’s instructions, where the tetrapeptide substrate is labeled with the chromophore, *p*-nitroaniline (pNA). pNA is released from the substrate upon cleavage by caspase-8, and the free pNA exerts a yellow color, which can be measured with an ELISA reader at an absorbance of 405 nm [40]. In brief, cells were washed with cold PBS, resuspended in lysis buffer and left on ice for 15 min. The lysate was centrifuged at 16,000× *g* at 4 °C for 15 min. Then, the supernatant was incubated with caspase-8 substrate (Ac-IETD–pNA) at 37 °C for 2 h before the absorbance was detected at 405 nm.

### 4.9. Total Antioxidant Capacity Assay

Total antioxidant capacity was measured by the ferric reducing ability of plasma (FRAP) assay, according to the instruction of Beyotime Institute of Biotechnology. N2a cells transfected with the plasmid sh644 and treated with/without MSC were collected and washed three times with PBS. The cell lysate was centrifuged at 12,000× *g* at 4 °C for 15 min, and the supernatant was collected for further experiments. The FRAP working solution was prepared freshly by mixing tripyridyltriazine (TPTZ) dilution, detection buffer and TPTZ solution in a ratio of 10:1:1 (*v*/*v*/*v*). A 5-μL sample was mixed with 180 μL of FRAP working solution and kept for 5 min at 37 °C. The absorbance of the reaction mixture was measured at 593 nm. The standard curve was prepared using the different concentrations of FeSO_4_ standard solutions ranging from 0.15 to 1.50 mM. The activity was expressed by FeSO_4_ values, which were calculated using standard curves [41].

### 4.10. Statistical Analysis

Statistical significance was assessed by a two-tailed paired *t*-test. The level of significance was set at *p* < 0.05, *p* < 0.01 and *p* < 0.001).

## 5. Conclusions

The mechanism of Clu and MSC involved in the regulation of apoptosis was investigated in this paper. Interference vectors were constructed to specifically and efficiently decrease the intracellular expression of Clu in N2a and SH-SY5Y cells. *Clu*-knockdown decreased cell antioxidative capacity and the Bcl-2/Bax ratio, meanwhile increasing caspase-8 activity, leading to cell apoptosis. In micromolar MSC could reverse the changes caused by *Clu*-knockdown and, thus, maintained cell viability and inhibited cell apoptosis. It is suggested that the appropriate amount of supplemental selenium could inhibit apoptosis induced with the decrease of Clu expression. Results in this paper imply that proper Clu expression is essential for the growth of neurocytes, and MSC supplemented at proper doses helps to prevent neurocytes from apoptosis induced by *Clu*-knockdown, both of which are important in preventing the development of AD.

## Abbreviations

AD: Alzheimer’s disease
c-Clu: cytoplasm Clu
Clu: clusterin
Clu-1: pLKO–shRNA–Clu1
Clu-2: pLKO–shRNA–Clu2
CrmA: cytokine response modifier A
FRAP: ferric reducing ability of plasma assay
HEK293T cells: human embryonic kidney 293T cells
MSC: Se-methylselenocysteine
N2a cells: mouse neuroblastoma N2a cells
Nc: pLKO–shRNA–Nc
n-Clu: nuclear Clu
PD: Parkinson disease
pNA: *p*-nitroaniline
RNAi: RNA interference
Sc: pGPU–*Clu*–mus–shSc
s-Clu: secreted Clu
Se: selenium
sh488: pGPU–*Clu*–mus-488
sh577: pGPU–*Clu*–mus-577
sh644: pGPU–*Clu*–mus-644
sh1535: pGPU–*Clu*–mus-1535
shRNA: short-hairpin RNAs
SH-SY5Y cells: human neuroblastoma SH-SY5Y cells
siRNA: small interfering RNA
TGF-β: transforming growth factor β
TNF-α: tumor necrosis factor α
